# Non-REM Sleep Characteristics Predict Early Cognitive Impairment in an Aging Population

**DOI:** 10.3389/fneur.2019.00197

**Published:** 2019-03-13

**Authors:** Jacques Taillard, Patricia Sagaspe, Christian Berthomier, Marie Brandewinder, Hélène Amieva, Jean-François Dartigues, Muriel Rainfray, Sandrine Harston, Jean-Arthur Micoulaud-Franchi, Pierre Philip

**Affiliations:** ^1^USR CNRS 3413 SANPSY Sommeil, Addiction et NeuroPSYchiatrie, Bordeaux, France; ^2^SANPSY, USR 3413, Université Bordeaux, Bordeaux, France; ^3^CHU de Bordeaux, Pôle Neurosciences Cliniques, Bordeaux, France; ^4^PHYSIP, Paris, France; ^5^CMRR, CHU Bordeaux, Bordeaux, France; ^6^Bordeaux Population Health Center, INSERM U1219, Université de Bordeaux, Bordeaux, France

**Keywords:** sleep, EEG, slow-wave sleep, spindle, cognition, cognitive decline, aging, mild cognitive impairment

## Abstract

**Objective:** Recent research suggests that sleep disorders or changes in sleep stages or EEG waveform precede over time the onset of the clinical signs of pathological cognitive impairment (e.g., Alzheimer's disease). The aim of this study was to identify biomarkers based on EEG power values and spindle characteristics during sleep that occur in the early stages of mild cognitive impairment (MCI) in older adults.

**Methods:** This study was a case-control cross-sectional study with 1-year follow-up of cases. Patients with isolated subjective cognitive complaints (SCC) or MCI were recruited in the Bordeaux Memory Clinic (MEMENTO cohort). Cognitively normal controls were recruited. All participants were recorded with two successive polysomnography 1 year apart. Delta, theta, and sigma absolute spectral power and spindle characteristics (frequency, density, and amplitude) were analyzed from purified EEG during NREM and REM sleep periods during the entire second night.

**Results:** Twenty-nine patients (8 males, age = 71 ± 7 years) and 29 controls were recruited at T0. Logistic regression analyses demonstrated that age-related cognitive impairment were associated with a reduced delta power (odds ratio (OR) 0.072, *P* < 0.05), theta power (OR 0.018, *P* < 0.01), sigma power (OR 0.033, *P* < 0.05), and spindle maximal amplitude (OR 0.002, *P* < 0.05) during NREM sleep. Variables were adjusted on age, gender, body mass index, educational level, and medication use. Seventeen patients were evaluated at 1-year follow-up. Correlations showed that changes in self-reported sleep complaints, sleep consolidation, and spindle characteristics (spectral power, maximal amplitude, duration, and frequency) were associated with cognitive impairment (*P* < 0.05).

**Conclusion:** A reduction in slow-wave, theta and sigma activities, and a modification in spindle characteristics during NREM sleep are associated very early with a greater risk of the occurrence of cognitive impairment. Poor sleep consolidation, lower amplitude, and faster frequency of spindles may be early sleep biomarkers of worsening cognitive decline in older adults.

## Introduction

Western societies are marked by aging of the general population which favors the increasing prevalence of neurological and sleep disorders. These disorders contribute to the morbidity and the mortality of the general population, in particular through daily life activities.

Recent evidence suggests that sleep disorders or modifications in sleep stage or electroencephalogram (EEG) waveform precede over time the onset of the clinical signs of mild cognitive impairment (MCI). This may be viewed as a transitional stage from normal cognition to dementia and Alzheimer's disease (AD), a neurodegenerative disorder characterized by progressive decline in memory and other cognitive domains.

A study (1) suggested that older adults suffering from sleep-disordered breathing, characterized by repeated episodes of hypoxemia and brief arousal, had a higher risk of developing long-term cognitive decline. An underlying mechanism of this relationship would seem to be hypoxemia rather than sleep fragmentation or sleep duration. However, a longitudinal study (2) showed that subsequent cognitive decline was associated with reduced sleep efficiency, greater wake after sleep onset (WASO), greater number of long wake episodes, and poor self-reported sleep quality. A recent study (3) showed that long sleep latency could also serve as an early marker of cognitive decline in MCI.

In addition to the sleep architecture or presence of sleep disorders, a new line of research is moving toward electroencephalogram oscillations as being involved in age-related cognitive decline.

The “Active System Consolidation theory of memory” posits that sleep, especially NREM sleep, promotes long-term consolidation of memories involving a dialog between the hippocampus and neocortex. (4–7) The key factor in hippocampal to neocortical transmission is the triple-phase that locks slow oscillations (cortex)-spindles (thalamus)-sharp wave-ripples (hippocampus) (7–9). Depolarizing slow oscillation up-states are involved in the generation of fast spindle (13–15 Hz) and sharp wave ripples, thus leading to the constitution of “spindle-ripple events” (9–11). Slow spindles (11–13 Hz) coincide with the slow oscillation up to down state transition but their role in memory consolidation is less well-documented. On the other hand, slow spindles are accompanied by an increase in lower frequencies, especially in the 5–8 Hz theta range (11). Theta oscillations during NREM might also be involved in strengthening memories (12). A study (13) demonstrated that reactivation of memory not only occurred in synchrony with spindles but was modulated by spindle amplitude: the higher amplitude, the stronger was the activity in the hippocampus.

There is growing evidence that sleep spindles, especially spindle characteristics (frequency, density, and/or amplitude), participate in memory formation, learning and synaptic plasticity (9, 14, 15).

The synaptic homeostasis hypothesis postulates that NREM sleep, especially slow wave sleep, restores synaptic, and cellular homeostasis that has been potentiated toward saturation during wakefulness (16). This theory predicts that the process of synaptic renormalization during sleep increases the capacity to acquire information during the following day (16, 17).

Healthy aging is accompanied by changes in sleep quantity, especially a decreased total sleep time, increased WASO, and decreased sleep efficiency (18, 19).

A highly important change in sleep with aging is a reduction in slow-wave sleep and in EEG slow-wave activity (18, 20–23). Sigma activity during NREM is decreased and the number, density and amplitude of sleep spindles are also reduced (23, 24).

A meta-analysis on sleep in patients with MCI showed less total sleep time and sleep efficiency and longer sleep latency (25). Another study (26) in patients with amnestic MCI demonstrated that slow wave sleep, delta, and theta power during NREM sleep were dramatically reduced. REM sleep, REM latency and sleep efficiency were also reduced. Spindle density, especially among fast spindles, was reduced. A recent study confirmed that parietal fast spindle density is decreased in MCI patients (27). As MCI progresses to Alzheimer's disease, sleep disturbances worsen. The amount of SWS is greatly reduced and spindles diminish in frequency (28–31) especially fast spindles (27, 29). The development of dementia in Parkinson patients was linked with sleep spindle density in posterior regions and sleep spindle amplitude in parietal regions. Lower sleep spindle amplitude in posterior regions was associated with poorer visuo-spatial abilities in patients who developed dementia at follow-up (32). The K-complex (KC) density during stage N2 decreases in AD patients but not in MCI patients (33, 34). KC density is positively correlated with cognitive deterioration. KC density cannot be considered as an early biomarker of AD but as a measure of cognitive decline (33). The reduction in KC density could reflect a dysfunction in synaptic plasticity linked with a deterioration in memory consolidation (34).

Slow oscillatory transcranial direct current stimulation in MCI patients led to enhanced endogenous slow wave-spindle coupling in the following way: spindle amplitude was significantly amplified during the depolarizing slow oscillation up-phases and synchronization between slow oscillation and fast spindle amplitude, involving an enhancement of visual declarative memory (35).

Moreover, alteration in sleep quantity, and quality facilitates the accumulation of amyloid-β, potentially initiating earlier cognitive decline and conversion to Alzheimer's disease. The disturbance of NREM sleep may represent a novel pathway though which cortical amyloid-β impairs hippocampus-dependent memory consolidation (31).

The aim of the SCOAL study is to determine polysomnographic biomarkers that occur at a very early stage of MCI. The objectives are the following:

- To compare the sleep architecture and/or presence of sleep disorders and neuropsychological performance in patients with isolated subjective cognitive complaints (SCC) or MCI, vs. cognitively normal controls.
- To study the prospective association of sleep and cognitive decline in patients with isolated SCC or MCI over a 1-year follow-up period.

## Methods

### Study Population

#### Patients

**Figure 1:**
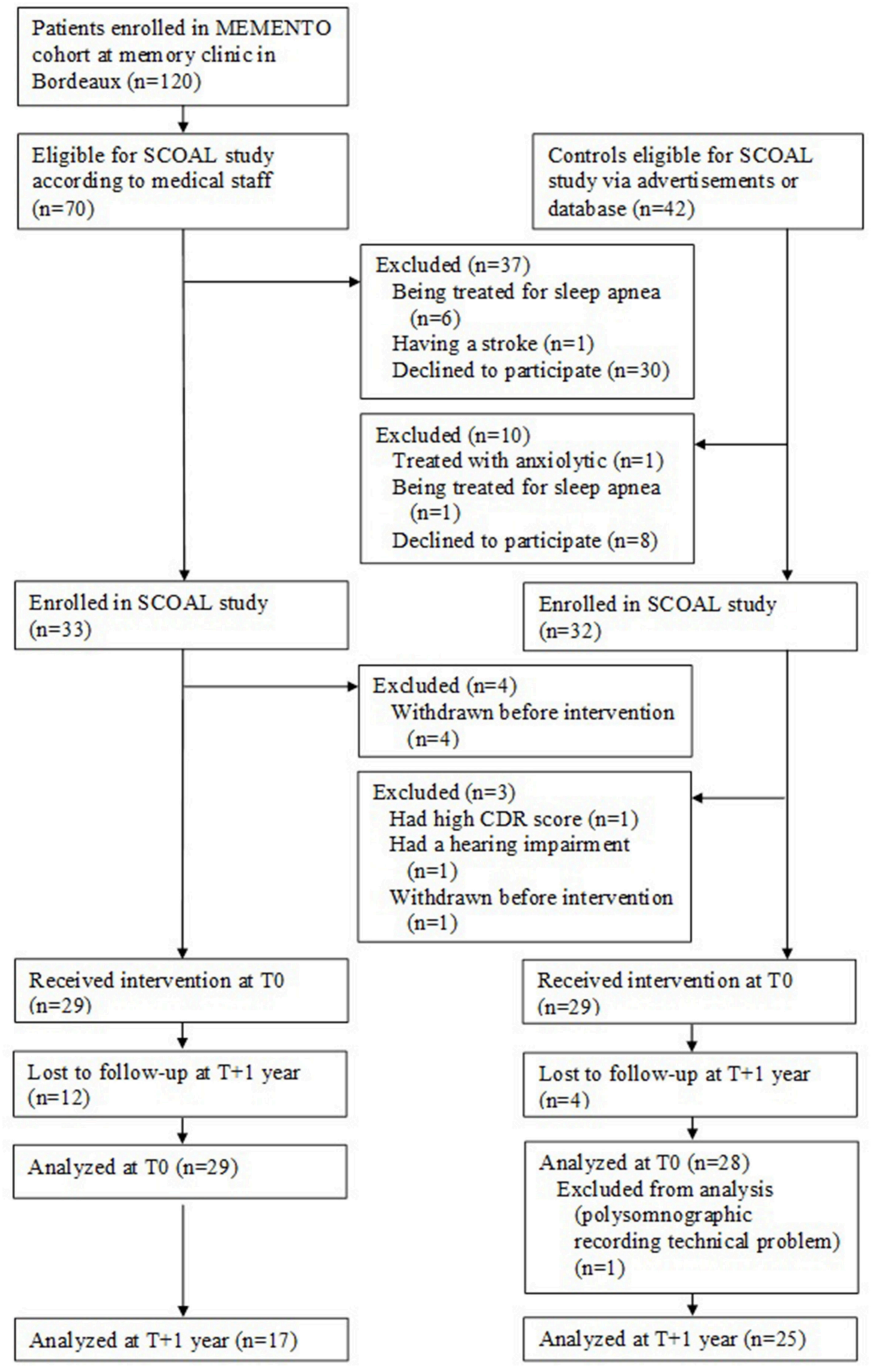
Flow charts SCOAL study.

The patients in the SCOAL study were recruited from the MEMENTO cohort at the University Hospital of Bordeaux from October 2011 to October 2015. MEMENTO (deterMinants and Evolution of AlzheiMer's disEase aNd relaTed disOrders) is a 5-year prospective large cohort of patients with either isolated SCC or recently diagnosed MCI while not demented attending an outpatient memory clinics (CMRR—Center Mémoire de Ressource et de Recherche) of public hospitals in France (36). The medical staff from the Bordeaux Memory Clinic invited eligible patients to participate in the SCOAL protocol (see Figure 1), which involved a 2-day stay in hospital and neuropsychological testing on attentional and executive functions (not detailed here).

MEMENTO inclusion criteria were as follows:

- Having at least MCI defined by a performance of more than 1.5 standard deviation from the mean (defined according to age, gender and level of education) in one or more cognitive domains (assessed from a battery of neuropsychological tests exploring memory, language, praxis, vision, executive functions), the deficit being identified for the first time by tests performed <6 months before the inclusion date;
- Or presenting an isolated cognitive complaint if the participant was over 60 years old;
- Having a clinical dementia rating [CDR] (37) Scale score ≤ 0.5 and being non-demented (DSM-IV).

As part of the MEMENTO study, participants completed a battery of neuropsychological tests administered by a trained neuropsychologist. In particular, the Mini-Mental State Examination (MMSE), which tests global cognition (38), the Free and Cued Selective Reminding Test (FCSRT) (39), the Trail-Making Test (TMT) (40) and the CDR (37) were administered. To assess the presence and severity of neuropsychiatric symptoms, the Neuropsychiatric Inventory (NPI) was used.

MCI was diagnosed using the Petersen Criteria (41).

The specific inclusion criteria for patients in the SCOAL study were as follows:

- Being at least 18 years old
- Having stable health (i.e., no medical condition involving imminent care or hospitalization)
- Being treated for obstructive sleep apnea syndrome (OSAS), if diagnosed.

#### Controls

Healthy controls considered cognitively normal at baseline and matched on age, gender, and level of education with patients underwent clinical and cognitive assessment. To verify the absence of objective cognitive deficit, participants were administered the MMSE (38), FCSRT (39), TMT (40), and CDR (37) by a trained neuropsychologist.

### Design

The 1-year follow-up SCOAL study was a case-control cross-sectional study with follow-up at 1 year.

Patients with isolated SCC or MCI from the MEMENTO cohort in Bordeaux and cognitively normal controls were recruited. The controls with patients were matched on age, gender, and level of education.

Participants were recorded with 2-night polysomnography (PSG) monitoring and were tested on a battery of neuropsychological tests assessing attention and executive functions at T0 and T+1 year.

To determine usual sleep patterns, volunteers' sleep was recorded via an actimeter over 3 days in a natural environment.

To assess sleep disorders, participants underwent a first night of polysomnographic sleep recording (PSG) at the hospital. Questionnaires were used to capture sleep complaints and excessive daytime sleepiness.

The participants also completed a battery of neuropsychological tests.

The second night of PSG recording was used for sleep and EEG analysis.

Information on current pathologies and medical treatment was also collected.

All participants gave written informed consent. The study was approved by the local ethical committee (consultative committee for the protection of persons participating in biomedical research, CPP Sud-Ouest et Outre Mer III). The study was registered with ClinicalTrials.gov, identifier: NCT01650454.

#### Neuropsychological Evaluation

Patients completed their neuropsychological evaluation as part of their cognitive follow-up at the Memory Clinic, at T0 and then after 1 year of follow-up. Controls completed a neuropsychological evaluation at the time of the inclusion visit.

Subjective cognitive complaints (SCC) were assessed by visual analog scales ranging from 0 to 10 on several domains (e.g., memory, attention, language).

The Mini-Mental State Examination (MMSE) (42) is one of the most widely used psychometric tests for quantifying global cognitive functioning and cognitive change in population-based longitudinal studies (total score, range 0–30).

The free and cued selective reminding test (FCSRT) (39) evaluates the ability to learn a list of 16 written words that are presented with a semantic cue to control for memory encoding. It distinguishes between simple difficulties in retrieval of stored information (facilitated by cuing) and genuine storage deficits characterizing typical Alzheimer's disease (not facilitated by cuing). The learning phase is followed by three trials of recall, each consisting in retrieving the words first spontaneously (“free recall”) and then with the help of a semantic cue (“cued recall”) for those items not retrieved by free recall. The three measures evaluated were free recall (the cumulative sum of free recall from the three trials; range 0–48), total recall (the cumulative sum of free recall + cued recall from the three trials, range 0–48), and delayed recall (sum of delayed free recall + delayed cued recall, range 0–16).

The Trail-Making Test (TMT) (43) is a two-part paper and pencil neuropsychological test which assesses executive function. Participants are required to connect numbered circles in a sequential order in the TMT-A, whereas they have to connect numbered and lettered circles in alternating sequential-alphabetical order in the TMT-B (i.e., 1-A-2-B, etc.). TMT-A time is taken as a measure of processing speed, while TMT-B time is considered an index of flexibility. The dependent variables are the number of seconds needed to complete the sequence and the number of correct responses for Part B and an interference index (Part A/Part B).

The Clinical Dementia Rating (CDR) (37) is a global scale developed to clinically denote the presence of dementia of the Alzheimer type and stage its severity. The clinical protocol incorporates semi-structured interviews with the patient and informant to obtain information necessary to rate the SCC in six domains (memory, orientation, judgment and problem solving, community affairs, home and hobbies, and personal care). The score is the CDR Scale Sum of Boxes (CDR-SOB).

### Self-Reported Sleep Measures

Participants completed questionnaires during the follow-up visits.

The Epworth Sleepiness Scale (ESS) (44), a self-administered questionnaire, is used to measure subjective daytime sleepiness. Scores range from 0 to 24, a score of >10 indicating excessive daytime sleepiness.

The Pittsburgh Sleep Quality Index (PSQI) (45) is a 19-item questionnaire assessing subjective sleep quality and disturbances over the past month. Scores range from 0 to 21, a score of >5 indicating poor sleep quality.

The Insomnia Severity Index (ISI) (46) is a seven-item questionnaire assessing the nature, severity and impact of insomnia symptoms over the past month. The total score ranges from 0 to 28.

### Objective Sleep Measures

#### Actigraphy

Participants had 3 days of monitoring with actimeters (Actiwatch, Cambridge Neurotechnology, Cambridge, United Kingdom). To study disrupted daily activity/rest cycles (47), the criteria assessed were as follows:

- Inter-daily stability (IS): measure of stability across days.
- Intra-daily variability (IV): relative consolidation within days.
- Rhythm amplitude (RA): difference in activity level between the 10 most active and five least active hours in the day.

#### Polysomnography

Participants slept two successive nights at the sleep unit. Polysomnographic signals were recorded with a Braintronics Brainbox EEG-1042 digital sleep recorder (Almere, The Netherlands, resolution 16-bit, stop band frequency 100 Hz, passband ripple 0.027 dB, stopband ripple −40 dB) at a sampled rate of 256 Hz. Nine Ag-AgCl electrodes were placed according to the international 10–20 System (F4, C4, O2, F3, C3, O1, Fz, Cz, Pz, Oz, M1, and M2) and referenced to linked-mastoids. Additionally, an electro-occulogram (EOG), an electromyogram (EMG, chin), and an electrocardiogram (ECG) were recorded as recommended by AASM (48) for the routine scoring of polysomnography. During the first night, nasal pressure, rib cage and abdominal movements, snoring sounds, transcutaneous finger pulse oximeter, and leg movements were also recorded. The first night was for adaptation and to identify the presence of any organic sleep disorders, while the second night was used for sleep and EEG analysis. Following the application of a notch filter (50 Hz) and a band pass filtered at 0.53–35 Hz, sleep stages and associated events were visually scored according to standard procedures (48) by the same experienced technician. The analyses of sleep EEG recordings were performed after an automatic artifact rejection using the ASEEGA software (version 3.30.14, Physip, France) (49–52). Artifacts are detected automatically based on both temporal and frequential criteria, where non-physiological abrupt variations are discarded. EEG spectral power per 30 s artifact-free epoch (Cz-Oz) was calculated using the fast Fourier transform with the Hanning window after an automatic artifact rejection. The spectral power was computed in the frequency bands delta (0.1–4 Hz), theta (4–8 Hz), alpha (8–12 Hz), sigma (12–16 Hz), and beta (16–50 Hz), for all the automatically scored R and NREM epochs. After their automatic detection, the spindles were characterized by their duration (s), power (squared microvolts), maximum amplitude (microvolts), and frequency (Hz) [for more details on spindle detection method see (53)]. The density of spindles was computed as the average number of detected spindles per 30 s epoch for each subject. The spindle maximal amplitude is measured on the EEG filtered in the sigma frequency band and corresponds to the maximum of the filtered signal envelope. Spindles falling within the 11–13 Hz frequency range were considered as “slow spindles,” and those falling in the 13–15 Hz range as “fast spindles” (27, 54). In addition, delta, theta, alpha, and beta spectral power and variations in spindle activity were computed for each successive NREM sleep cycles as defined by Feinberg and Floyd criteria (55).

## Statistical Analyses

Quantitative variables were expressed as mean ± standard deviation (*SD*), and qualitative variables were expressed as relative frequency.

### Cross-Sectional Analyses at T0

First, univariate analyses with *t*-test comparisons for continuous variables or chi-square tests (χ^2^) for categorical variables were used to compare demographic, clinical, neuropsychological, polysomnographic and log-transformed EEG characteristics in subjects with isolated SCC or MCI, with those characterized as cognitively normal.

Variables were expressed as continuous variables or as categorical variables as follows:

Age; Gender; Body mass index (BMI); Educational level; Self-reported pathologies: diabetes or hypertension; Self-reported current medication use: antidepressants, benzodiazepines or non-benzodiazepine anxiolytics; Neuropsychological performance: MMSE score, number of words at Free recall, Total recall and Delayed recall, TMT-B scores, TMT interference index; CDR score; ESS score; PSQI score; ISI score; Actimetric parameters: inter-daily stability (IS), intra-daily variability (IV), rhythm amplitude (RA); Polysomnographic characteristics: Time in bed (TIB); Total sleep time (TST); Sleep structure: Sleep latency, Stages N1, N2, N3, REM, sleep efficiency; Wake after sleep onset (WASO-PTS): >58, index of micro-arousals: >20 events/h; Apnea/Hypopnea Index (AHI): >10 events/h, >30 events/h; Apnea/Hypopnea central index; Periodic limb movements (PLM) index: >15 events/h; Respiratory effort-related arousals index (RERAs); Respiratory disorder index (RDI); Snoring; Mean SaO2; Minimum SaO2; SaO2 <90%; Oxygen desaturation index.

Then, multivariate analyses with logistic regression models were conducted to control for potential confounding factors on the association between sleep parameters and cognitive impairment. Multivariate models were adjusted on age, gender, BMI, education level, use of antidepressant, benzodiazepine and non-benzodiazepine anxiolytics.

Results are presented as odds ratios (ORs) with 95% confidence intervals (CIs).

The alpha risk threshold was set at *P* = 0.05.

### Longitudinal Analyses

Yearly changes in self-reported and objective sleep variables and cognitive variables were computed as the subtraction of the T0 score from the T+1-year score for individual performance.

Then, non-parametric correlations (Spearman) were computed for patients between sleep and cognitive variables scores.

All statistical analyses were performed using the SPSS statistical software package (PASWR Statistics 18).

## Results

### Characteristics of Patients vs. Controls at T0

#### Population

Twenty-nine patients with isolated SCC or MCI enrolled from the MEMENTO cohort received the intervention at T0 (see Figure 1).

Concerning cognitive status, 21% had amnestic MCI (17% single-domain amnestic MCI), 48% non-amnestic MCI (79% single-domain non-amnestic MCI) and 31% had isolated SCC.

Twenty-nine matched controls characterized as cognitively normal were recruited after the clinical and neuropsychological examination at baseline (see Figure 1).

#### Demographic Characteristics

Concerning patients, most were female (*n* = 21; 72.4%), they had a mean age of 71 (*SD* = 7) years (range: 58–85 years), a mean BMI of 24.1 (*SD* = 3.9), and an education level of 12.4 (*SD* = 3.8) years. 69% had graduated from high school and attended university.

The latter were matched on age, gender, and educational level with cognitively normal controls. Most were female (*n* = 21; 72.4%), had a mean age of 68.1 ± 4.4 years (range: 58–77 years) and an education level of 11.8 ± 4.2 years. 48.3% had graduated from high school and attended university.

Participants with isolated SCC or MCI did not differ from cognitively normal controls on demographic parameters (see Table 1).

**Table 1:** Demographic, clinical, neuropsychological and polysomnographic characteristics (Mean ± SD) in patients with isolated subjective cognitive complaints or mild cognitive impairment, and in cognitively normal controls, at first intervention (T0). Statistical significance (*P* values) for independent groups with *T*-tests for continuous variables or Chi-square test (χ^2^) for categorical variables.

	**Patients with isolated cognitive complaints or mild cognitive impairment T0 (*n* = 29)**	**Controls T0 (*n* = 29)**	***P* value**
**DEMOGRAPHIC AND CLINICAL CHARACTERISTICS**			
Age (years)	71.0 ± 7.0	68.1 ± 4.4	ns
Gender (% females)	72.4	72.4	ns
Body Mass Index (BMI) (kg/m^2^)	24.1 ± 3.9	23.9 ± 4.3	ns
Educational level (years)	12.4 ± 3.8	11.8 ± 4.2	ns
**PATHOLOGIES** (**%**)			
Diabetes	0.0	3.5	ns
Hypertension	15.8	8.8	ns
**CURRENT MEDICATION USE** (**%**)			
Antidepressants	12.3	0.0	<0.01
Benzodiazepines	3.5	1.8	ns
Non-benzodiazepine anxiolytics	1.8	0.0	ns
**MINI-MENTAL STATE EXAMINATION (MMSE)**			
Score	28.1 ± 1.5	28.2 ± 1.6	ns
≥24	100%	100%	ns
**FREE AND CUED SELECTIVE REMINDING TEST**			
Free recall	29.1 ± 6.8	31.5 ± 6.9	ns
Total recall	45.3 ± 5.4	46.1 ± 2.5	ns
Delayed recall	15.6 ± 1.2	12.3 ± 2.5	<0.001
**TRAIL-MAKING TEST (TMT)**			
TMT-B (RTs)	96.9 ± 40.8	107.1 ± 79.0	ns
TMT-B (correct)	22.5 ± 4.3	22.4 ± 2.4	ns
TMT interference index (RTs)	0.5 ± 0.1	0.4 ± 0.2	ns
**CLINICAL DEMENTIA RATING (CDR)**			
	0.40 ± 0.21	0.01 ± 0.09	<0.001
**SELF-REPORTED QUESTIONNAIRES**			
Epworth Sleepiness Scale (ESS) score	8.6 ± 4.7	6.8 ± 3.9	ns
>10	34%	17%	
Pittsburgh Sleep Quality Index (PSQI)	7.4 ± 3.8	6.8 ± 3.6	ns
>5	66%	55%	
Insomnia Severity Index (ISI)	8.7 ± 5.1	8.9 ± 6.5	ns
≥15	17%	21%	
**ACTIMETRIC PARAMETERS**			
Inter-daily stability (IS)	0.73 ± 1.09	0.70 ± 0.10	ns
Intra-daily variability (IV)	0.77 ± 0.23	0.82 ± 0.20	ns
Rhythm amplitude (RA)	0.92 ± 0.05	0.93 ± 0.03	ns
**POLYSOMNOGRAPHIC CHARACTERISTICS**			
Time in bed (min)	478 ± 19.8	472 ± 23.7	ns
Total sleep time (min)	382 ± 62.7	391 ± 52.4	ns
Sleep latency (min)	9.0 ± 9.0	10.6 ± 10.4	ns
Stage N1 (%)	7.1 ± 3.3	7.1 ± 4.4	ns
Stage N2 (%)	50.2 ± 11.2	48.8 ± 8.4	ns
Stage N3 (%)	20.5 ± 8.1	20.8 ± 10.0	ns
Stage REM (%)	22.1 ± 6.8	23.3 ± 5.2	ns
Sleep efficiency (%)	80.0 ± 13.0	82.7 ± 9.2	ns
Wake After Sleep Onset—PTS (WASO)	74.6 ± 57.7	51.8 ± 29.3	ns
>58 (min) (median)	58.6%	39.3%	ns
Index of micro-arousals	25.7 ± 13.2	25.6 ± 14.3	ns
>20 events/h (*n*)	65.5%	55.2%	ns
Apnea/Hypopnea Index (AHI)	19.1 ± 14.1	19.4 ± 13.6	ns
>10 events/h (*n*)	72.4%	65.5%	ns
>30 events/h (*n*)	13.8%	20.7%	ns
Apnea/Hypopnea Central Index	0.24 ± 1.1	1.25 ± 6.4	ns
Periodic Limb Movements (PLM) Index	16.6 ± 20.3	9.2 ± 15.9	ns
>15 events/h (n)	37.9%	17.2%	ns
Respiratory Effort Related Arousals Index (RERAs)	1.25 ± 1.8	0.81 ± 1.3	ns
Respiratory Disorder Index (RDI)	20.3 ± 13.9	20.2 ± 19.6	ns
Snoring	5.5 ± 10.2	2.2 ± 4.8	ns
Mean SaO2 (%)	92.9 ± 2.2	93.0 ± 1.4	ns
Minimum SaO2 (%)	83.7 ± 7.2	85.9 ± 5.4	ns
SaO2 <90% (*n*)	47.1 ± 88.5	30.1 ± 41.4	ns
Oxygen desaturation index, events/h	19.2 ± 16.9	17.1 ± 14.0	ns

*SD, Standard Deviation; n, Number; SaO2, Oxygen saturation*.

#### Clinical Characteristics

Regarding cardiovascular risk factors, there was no difference in the proportion of individuals suffering from diabetes or hypertension between those with isolated SCC or MCI and the controls.

Regarding medication use, a significantly higher proportion of patients with isolated SCC or MCI used antidepressants (12.1%) compared to controls (0%) (*P* < 0.01). There was no difference in the proportion of patients with isolated SCC or MCI and controls regarding benzodiazepine or non-benzodiazepine anxiolytic use (see Table 1).

Regarding the baseline neuropsychological evaluation, the CDR score was higher in patients with isolated SCC or MCI than in controls (see Table 1).

Patients with isolated SCC or MCI do not report more excessive daytime sleepiness, or insomnia complaints than controls.

#### Actimetric Parameters

There was no difference in actimetric data between patients with isolated SCC or MCI and controls (see Table 1).

#### EEG Characteristics (PSG)

The PSG was not correctly recorded in one patient.

Out of 28 patients, 11 presented at least 4 NREM sleep periods (vs. 18 controls) and 24 presented at least 3 NREM sleep periods (vs. 26 controls) during their PSG recording.

##### Macro-architecture of sleep

Two-thirds to three-quarters of the participants (65.5% of controls and 72.4% of patients, respectively) met the criteria for sleep-disordered breathing with an AHI of 10 or more events per hour.

37.9% met the criteria for PLM disorder with an index of 15 or more events per hour vs. 17.2% in controls (*P* = 0.08).

There was no difference in sleep structure (% stage 1, 2, 3, and REM), sleep duration (TST), sleep propensity (sleep onset latency) between patients with isolated SCC or MCI and controls (see Table 1).

Regarding sleep consolidation parameters, the mean WASO was of greater duration in patients with isolated SCC or MCI (74.6 ± 57.7 min) than in cognitively intact controls (51.8 ± 29.3 min, *P* = 0.07, tendency). There was no difference in sleep efficiency between patients with isolated SCC or MCI and controls.

##### Micro-architecture of sleep

**Figure 2:**
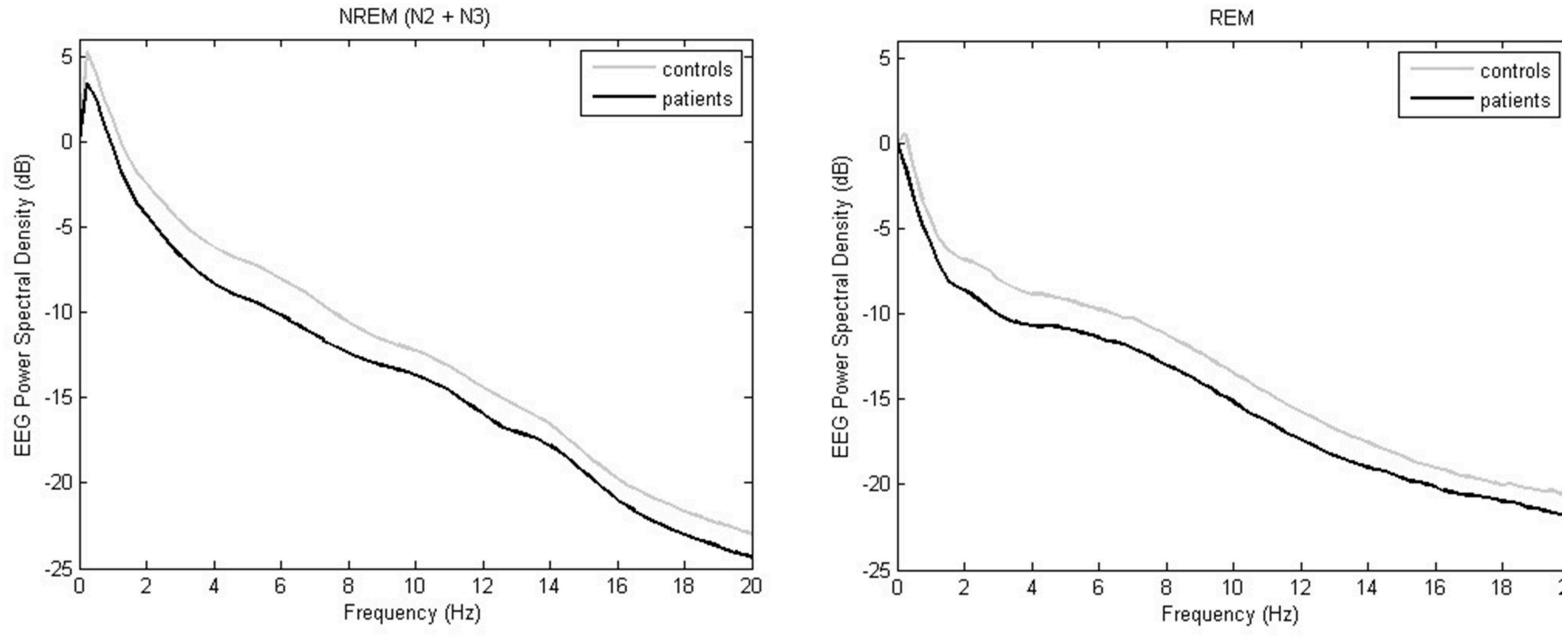
Whole-night EEG power spectral densities during NREM sleep and REM sleep in controls (gray) or SSC and MCI patients (black). Mean absolute values (expressed in logarithmic scale) are plotted in the frequency range from 0 to 20 Hz for 0.25 Hz bins.

Delta power, theta power and sigma power during NREM sleep periods were lower in patients with isolated SCC or MCI than in cognitively normal controls (see Table 2, Figure 2).

**Table 2:** Log-transformed EEG characteristics (Mean ± SD), in patients with isolated subjective cognitive complaints or mild cognitive impairment, and in cognitively normal controls, at first intervention (T0).

	**Patients with isolated cognitive complaints or mild cognitive impairment T0 (*n* = 29)**	**Controls T0 (*n* = 29)**	***P* value**
**EEG CHARACTERISTICS**			
**Spindle spectral power**			
During total stage 2	1.247 ± 0.281	1.420 ± 0.291	<0.05
In stage 2 cycle 1	1.309 ± 0.285	1.466 ± 0.309	=0.051
In stage 2 cycle 2	1.240 ± 0.281	1.392 ± 0.290	=0.053
In stage 2 cycle 3	1.228 ± 0.279	1.389 ± 0.299	=0.052
In stage 2 cycle 4	1.143 ± 0.342	1.374 ± 0.292	ns
During total stage 3	1.112 ± 0.270	1.257 ± 0.287	=0.055
In stage 3 cycle 1	1.197 ± 0.224	1.322 ± 0.293	ns
In stage 3 cycle 2	1.097 ± 0.302	1.241 ± 0.282	ns
In stage 3 cycle 3	1.046 ± 0.281	1.235 ± 0.284	<0.05
In stage 3 cycle 4	1.005 ± 0.384	1.182 ± 0.304	ns
**Spindle maximal amplitude**			
During total stage 2	0.915 ± 0.137	1.003 ± 0.142	<0.05
In stage 2 cycle 1	0.947 ± 0.138	1.027 ± 0.152	<0.05
In stage 2 cycle 2	0.912 ± 0.135	0.993 ± 0.140	<0.05
In stage 2 cycle 3	0.907 ± 0.137	0.987 ± 0.148	<0.05
In stage 2 cycle 4	0.866 ± 0.167	0.983 ± 0.141	=0.052
During total stage 3	0.847 ± 0.133	0.923 ± 0.142	<0.05
In stage 3 cycle 1	0.883 ± 0.104	0.957 ± 0.144	<0.05
In stage 3 cycle 2	0.844 ± 0.141	0.916 ± 0.138	ns
In stage 3 cycle 3	0.810 ± 0.133	0.912 ± 0.140	<0.05
In stage 3 cycle 4	0.782 ± 0.177	0.901 ± 0.145	ns
**Spindle duration**			
During total stage 2	−0.078 ± 0.031	−0.073 ± 0.027	ns
In stage 2 cycle 1	−0.082 ± 0.039	−0.081 ± 0.042	ns
In stage 2 cycle 2	−0.078 ± 0.028	−0.077 ± 0.029	ns
In stage 2 cycle 3	−0.080 ± 0.040	−0.071 ± 0.031	ns
In stage 2 cycle 4	−0.077 ± 0.032	−0.072 ± 0.035	ns
During total stage 3	−0.098±−0.042	−0.099 ± 0.031	ns
In stage 3 cycle 1	−1.107 ± 0.040	−1.008 ± 0.041	ns
In stage 3 cycle 2	−0.101 ± 0.059	−0.100 ± 0.053	ns
In stage 3 cycle 3	−0.101 ± 0.055	−0.082 ± 0.078	ns
In stage 3 cycle 4	−0.093 ± 0.054	−0.066 ± 0.061	ns
**Spindle frequency**			
During total stage 2	1.136 ± 0.023	1.130 ± 0.018	ns
In stage 2 cycle 1	1.135 ± 0.023	1.129 ± 0.018	ns
In stage 2 cycle 2	1.136 ± 0.023	1.130 ± 0.017	ns
In stage 2 cycle 3	1.133 ± 0.023	1.130 ± 0.017	ns
In stage 2 cycle 4	1.136 ± 0.022	1.126 ± 0.018	ns
During total stage 3	1.132 ± 0.023	1.126 ± 0.017	ns
In stage 3 cycle 1	1.131 ± 0.025	1.123 ± 0.016	ns
In stage 3 cycle 2	1.131 ± 0.023	1.127 ± 0.016	ns
In stage 3 cycle 3	1.130 ± 0.024	1.125 ± 0.023	ns
In stage 3 cycle 4	1.134 ± 0.026	1.124 ± 0.023	ns
**Spindle counts**			
Slow	0.468 ± 0.759	0.499 ± 0.665	ns
Fast	1.793 ± 1.077	1.583 ± 0.959	ns
**NREM delta spectral power**			
During total NREM	2.365 ± 0.310	2.558 ± 0.321	<0.05
In NREM cycle 1	2.434 ± 0.347	2.488 ± 0.338	ns
In NREM cycle 2	2.272 ± 0.337	2.601 ± 0.371	<0.05
In NREM cycle 3	2.234 ± 0.299	2.491 ± 0.368	<0.05
In NREM cycle 4	2.091 ± 0.443	2.371 ± 0.370	ns
**NREM theta spectral power**			
During total NREM	1.290 ± 0.280	1.497 ± 0.219	<0.05
In NREM cycle 1	1.327 ± 0.290	1.521 ± 0.262	<0.05
In NREM cycle 2	1.270 ± 0.290	1.496 ± 0.216	<0.05
In NREM cycle 3	1.294 ± 0.308	1.458 ± 0.227	<0.05
In NREM cycle 4	1.253 ± 0.355	1.509 ± 0.201	<0.05
**NREM alpha spectral power**			
During total NREM	0.936 ± 0.285	1.094 ± 0.235	<0.05
In NREM cycle 1	0.982 ± 0.289	1.119 ± 0.267	ns
In NREM cycle 2	0.938 ± 0.292	1.087 ± 0.243	<0.05
In NREM cycle 3	0.923 ± 0.286	1.094 ± 0.249	<0.05
In NREM cycle 4	0.864 ± 0.350	1.073 ± 0.207	ns
**NREM sigma spectral power**			
During total NREM	0.518 ± 0.264	0.672 ± 0.266	<0.05
In NREM cycle 1	0.560 ± 0.255	0.687 ± 0.285	ns
In NREM cycle 2	0.523 ± 0.277	0.643 ± 0.272	ns
In NREM cycle 3	0.474 ± 0.268	0.677 ± 0.289	<0.05
In NREM cycle 4	0.460 ± 0.315	0.652 ± 0.258	ns
**NREM beta spectral power**			
During total NREM	0.437 ± 0.280	0.524 ± 0.227	ns
In NREM cycle 1	0.431 ± 0.262	0.519 ± 0.240	ns
In NREM cycle 2	0.387 ± 0.282	0.484 ± 0.236	ns
In NREM cycle 3	0.341 ± 0.290	0.537 ± 0.249	<0.05
In NREM cycle 4	0.317 ± 0.315	0.491 ± 0.233	ns
**REM theta spectral power**			
During total REM	1.150 ± 0.323	1.321 ± 0.245	<0.05
In REM cycle 1	1.154 ± 0.318	1.330 ± 0.251	<0.05
In REM cycle 2	1.176 ± 0.336	1.340 ± 0.254	<0.05
In REM cycle 3	1.159 ± 0.331	1.307 ± 0.266	ns
In REM cycle 4	1.107 ± 0.424	1.344 ± 0.229	ns
**REM alpha spectral power**			
During total REM	0.824 ± 0.327	1.007 ± 0.258	<0.05
In REM cycle 1	0.811 ± 0.318	1.016 ± 0.269	ns
In REM cycle 2	0.850 ± 0.355	1.047 ± 0.267	<0.05
In REM cycle 3	0.854 ± 0.324	1.000 ± 0.276	ns
In REM cycle 4	0.828 ± 0.381	0.971 ± 0.229	ns
**REM beta spectral power**			
During total REM	0.591 ± 0.319	0.731 ± 0.302	ns
In REM cycle 1	0.629 ± 0.340	0.770 ± 0.301	ns
In REM cycle 2	0.606 ± 0.316	0.728 ± 0.311	ns
In REM cycle 3	0.551 ± 0.320	0.729 ± 0.318	=0.052
In REM cycle 4	0.483 ± 0.285	0.635 ± 0.247	ns

*Statistical significance (P values) for independent groups with T-tests for continuous variables*.

*SD, Standard Deviation*.

Theta power and alpha power during REM sleep periods were lower in patients with isolated SCC or MCI than in cognitively normal controls (see Table 2, Figure 2).

Regarding spindle parameters across NREM, spindle maximal amplitude was lower in patients with isolated SCC or MCI in the four NREM sleep periods than in controls. There was no difference in spindle density, duration and frequency between the groups (see Table 2).

#### Association Between Cognitive Impairment and Sleep Parameters

Logistic regression analyses showed that a reduced spindle maximal amplitude (OR = 0.002, *p* < 0.05), delta power (OR = 0.072, *p* < 0.05), theta power (OR = 0.018, *p* < 0.01), and sigma power (OR = 0.033, *p* < 0.05) during NREM sleep periods were risk factors associated with isolated SCC or MCI in aging. Variables were adjusted on age, gender, BMI, educational level, and medication use (see Table 3).

**Table 3:** Multivariate logistic regression results for predicting isolated subjective cognitive complaints or mild cognitive impairment in older volunteers at first intervention (T0). Figures are adjusted odds ratios and 95% confidence intervals (CI) for multivariate model.

	**Odds ratio (95 %CI)**	***P*-value**
**SPINDLE MAXIMAL AMPLITUDE**			
During total stage 2	**0.002** (0.000–0.354)	<0.05
**NREM DELTA SPECTRAL POWER**			
During total NREM	**0.072** (0.008–0.621)	<0.05
**NREM THETA SPECTRAL POWER**			
During total NREM	**0.018** (0.001–0.321)	<0.01
**NREM SIGMA SPECTRAL POWER**			
During total NREM	**0.033** (0.002–0.527)	<0.05

*Multivariate models were adjusted on age, gender, BMI, education level, use of antidepressant, benzodiazepine, and non-benzodiazepine anxiolytics*.

*CI, Confidence Intervals; BMI, Body Mass Index; NREM, Non-Rapid Eye Movement*.

Bold values correspond to Odds ratio (no unit)

### One-Year Follow-Up Analyses

Seventeen patients were evaluated in follow-up.

Concerning their cognitive status, 35% had amnestic MCI (33% single-domain aMCI), 35% non-amnestic MCI (67% single-domain naMCI), and 30% had isolated SCC.

Considering decline at group level in patients with isolated SCC or MCI, there was no difference in neuropsychological performance during the 1-year follow-up.

Considering decline at an individual level, there were significant correlations between changes on self-reported or objective sleep parameters and cognitive impairment in patients with isolated SCC or MCI during the 1-year follow-up (see Table 4).

**Table 4:** Spearman correlations between yearly changes (subtraction of T0 score from T+1-year score for individual performance) on neuropsychological and polysomnographic and log-transformed EEG characteristics in patients with isolated subjective cognitive complaints or mild cognitive impairment.

**Yearly Changes**	**PSQI**	**ISI**	**Sleep efficiency**	**WASO**	**Fast spindle (counts)**	**Slow spindle (counts)**	**Spindle spectral power (during total stage 3)**	**Spindle maximal amplitude (during total stage 2)**	**Spindle maximal amplitude (during total stage 3)**	**Spindle duration (during total stage 2)**	**Spindle frequency (during total stage 2)**	**Spindle frequency (during total stage 3)**
**MINI-MENTAL STATE EXAMINATION (MMSE)**												
Score							0.56^*^	0.50^*^	0.62^*^			
**FREE AND CUED SELECTIVE REMINDING TEST**												
Free recall Total recall Delayed recall	−0.67^**^	−0.50^*^	0.53^*^	−0.51^*^						0.51^*^		
**TRAIL MAKING TEST (TMT)**												
TMT-B (RTs) TMT-B (correct) TMT interference Index (RTs)					0.63^**^	−0.67^**^ 0.48^*^					0.66^**^	0.56^*^

*RTs, reaction times; PSQI, Pittsburgh Sleep Quality Index; ISI, Insomnia Severity Index; WASO, Wake After Sleep Onset—PTS*.

* *P < 0.05*,

** *P < 0.01*.

MMSE score was positively correlated with EEG spindle amplitude during NREM, with higher cognitive degradation for 1 year associated with smaller spindle amplitude.

FCSRT (total recall) was correlated with awakenings during the night measured by sleep efficiency with higher memory degradation associated with weaker sleep consolidation.

TMT (TMT-B RTs) was correlated with EEG spindle frequency activity with higher impairment in executive function associated with faster spindle frequency or with a lower number of slow spindles or with a higher number of fast spindles.

## Discussion

To our knowledge, this study is the first to examine prospectively whether electroencephalogram (EEG) power values and sleep architecture measured by highly controlled in lab polysomnography are directly or indirectly informative of progressive cognitive decline in patients with isolated SCC or MCI.

The cross-sectional investigation (i.e., patients with isolated SCC or MCI/cognitively normal controls) demonstrates that during NREM sleep, a reduction in delta (slow wave), theta and sigma activities and in spindle maximal amplitude are associated very early with an increased risk of the occurrence of isolated SCC or MCI (25–32).

The present findings confirm that changes in NREM sleep patterns seem to be predisposing factors for the early onset of MCI. No difference was observed in sleep architecture, sleep apnea, or periodic limb movement indices between the groups.

Our results showed a marked decline in NREM and REM sleep EEG power in SSC and MCI patients. As reported previously (21, 22, 56), the decrease in power spectral density associated with normal aging was not limited to slow wave activity but also affected theta and sigma activity in NREM and in REM. These EEG modifications, especially low-frequency delta activity during both NREM and REM sleep, were associated with thinning of the frontal and prefrontal gray matter (56, 57). In contrast to our results and the change in EEG with age, Latreille et al. (58) observed that Parkinson's patients who developed dementia had a slowing of EEG in NREM sleep (higher power in delta and theta bands).

The 1-year follow-up demonstrates that the worsening cognitive decline in SCC/MCI patients is associated with changes in spindle characteristics (spectral power, maximal amplitude, and frequency) and with the impairment of sleep consolidation. In particular, a decrease in spindle maximal amplitude was associated with overall cognitive decline (i.e., MMSE), while an increase in spindle frequency was associated with a decline in executive functions. Moreover, changes in sleep consolidation parameters (i.e., sleep efficiency and WASO) together with changes in subjective sleep complaints (i.e., sleep quality and insomnia) were associated with episodic memory decline. Therefore, sleep consolidation and/or spindle characteristics (amplitude and frequency) could be early biomarkers that determine which SCC/MCI patients are at the greatest risk of suffering impaired cognitive or memory functions.

Sleep, especially slow-wave sleep plays an important role in the consolidation of long-term memory (5). Slow-wave activity reflects neural synchrony mainly within the prefrontal cortex, which may increase cortical connections that are important for cognition (57). A study has shown that sleep increases beta amyloid peptide (Aβ) clearance in interstitial fluid, promoting the removal of Aβ from the brain (59). Therefore, sleep disturbances, or modifications may be related to impairment of Aβ clearance and Aβ accumulation in the central nervous system, which leads to amyloid plaques, a characteristic of AD. Amyloid deposition in the preclinical stage of AD appears to be associated with worse sleep quality, especially sleep consolidation (60). Moreover, reduced SWS is associated with high cerebrospinal fluid Aβ in cognitively normal elderly (61). Finally, a recent study linked Aβ pathology with a reduction of SWS and the associated sleep-dependent memory consolidation, further supporting the existence of links between Aβ pathology, cognitive decline and sleep disturbances (62). Increased WASO and decreased SWS lead to relative increases in synaptic and metabolic neuronal activity, increased soluble CSF Aβ levels during the sleep period, increased Aβ aggregation, and sequestration into plaques, and attenuation of the Aβ diurnal pattern (63). Mander et al. (62) propose that sleep fragmentation and sleep efficiency quantified by actigraphy recorded during more than 10 days may be an early independent or complementary biomarker of AD risk. Although our results do not demonstrate that sleep fragmentation and efficiency differs between patients and controls, 1-year follow-up shows that more than a reduction in delta activity, it is above all the increase in wake time during sleep which is responsible for the worsening of memory in SCC/MCI patients.

In a recent study that examined the association between quantitative sleep EEG changes measured at home and clinical manifestations of MCI and/or incident dementia, ^64^ baseline EEG power values were higher in the group that developed dementia/MCI. Values were higher in the alpha and theta bands in NREM sleep, and in the alpha and sigma bands in REM sleep.

Like Djonlagic et al. (64), we did not find relationship between worsening of memory and modification in the delta bands during NREM. Either the association between EEG delta activity and cognition across the lifespan is more complex, reflecting an age-related dissociation of the functional relationship between delta activity and cognition, or it is the alteration of delta activity which precedes the clinical onset of cognitive decline.

Our results confirm that sigma activity and spindle characteristics are the marker of cognitive functioning in older adults. Like Djonlagic et al. (64), we found that the worsening of memory in MCI patients is not associated with a modification of sigma activity during NREM. On the other hand, we show that the worsening of memory in MCI patients is associated with a modification of spindle characteristics (amplitude and frequency). A decrease in spindle maximal amplitude is associated with overall cognitive deterioration (i.e., MMSE) and we confirm that spindle amplitude is positively correlated with cognitive ability (65).

We found that a higher density of fast spindles or an increase in spindle frequency during NREM were associated with poorer performance on executive function (i.e., mental flexibility). This result is in disagreement with one of the few studies to focus on changes in the density of spindle subtypes in association with overall cognitive decline (27). However, studies in children and adolescents have shown an association between slow spindle activity, learning efficiency, and general cognitive abilities (66) or a negative association between spindle activity and cognitive performance (67).

These results are seemingly at odds with findings in younger adults. However, Bang et al. (68) demonstrated that slow sigma activity corresponding to slow spindles was involved in the consolidation of a texture discrimination task. Further studies should investigate the relationships between sleep spindles, episodic memory, and overall cognitive abilities in aging and/or during life time. Moreover, the local specificity of alterations in slow and fast spindle activity and its relation to the severity of specific cognitive decline remain unclear.

An important question is whether slow wave activity or sleep spindle activity could be stimulated to prevent, or at least slow down, cognitive decline in the elderly. Slow oscillatory transcranial direct current stimulation in MCI (35) would be a good approach to improve the physiology of disordered sleep and memory deficits.

Sleep-disordered breathing is frequent among elderlies and is known to alter cognition in aging (69). We observed a high apnea/hypopnea index and high measures of sleep-related hypoxemia in both our groups. Therefore, the impact of sleep-disordered breathing on cognitive impairment could not be investigated here.

The strengths of this study are the examination of self-reported and objectively measured sleep parameters in the laboratory on several nights in the least severely affected patients of a cohort with isolated SCC or MCI. Moreover, we adjusted on potentially important confounding factors such as age, gender, BMI, education level, use of antidepressant, benzodiazepine, and non-benzodiazepine anxiolytics.

To overcome the limitations of our study, additional studies with larger sample sizes and a longer follow-up period involving clear clinical deterioration are required. Patients with SCC or MCI and controls suffered from psychiatric, cardiovascular, metabolic, and sleep (especially Obstructive Sleep Apnea Hypopnea) disorders and were receiving treatment (especially antidepressants). These disorders can modify sleep patterns and cognitive measures. We believe that the sleep EEG modifications or cognitive impairments in patients with SCC or MCI might not have been caused by these disorders, since there was no difference in the proportion of individuals suffering from these disorders between those with isolated SCC or MCI and the controls. Antidepressants can modify REM sleep parameters, but they did not differ between MCI patients and controls in this study. To account for these factors (pathologies and treatments), we adjusted the regression analyses. EEG was analyzed only at the CzOz localization, yet power activity and spindle characteristics were localized according to age. A study demonstrated that changes in sleep spindles related to age follow topographical patterns that are specific to each spindle characteristic, and that age-related changes in spindle density and frequency differ between men and women homogeneously across brain regions (70). Moreover, it would have been interesting to evaluate which of these patients will develop Alzheimer's disease in the future to determine whether sleep disturbances are key to an early diagnosis.

To conclude, our results demonstrate the following: (1) cognitive decline in SCC/MCI patients is associated with a reduction in slow-wave delta, theta and sigma (spindle) activities, and in spindle maximal amplitude during NREM sleep; (2) spindle characteristics (amplitude and frequency) and sleep consolidation parameters (sleep efficiency or WASO) could potentially serve as early sleep biomarkers for worsening cognitive decline with aging. Memory decline is associated with an increase in wake time during sleep. Overall cognitive decline is associated with a decrease in spindle amplitude, and the impairment of executive functions is associated with an increase in spindle frequency.

The findings of this study support the use of quantitative sleep EEG analysis as a promising biomarker for older people at risk of cognitive decline. An algorithm for the automatic detection of MCI and dementia markers in sleep EEG would be a welcome development.

Further research is necessary to unravel more precisely the associations between specific sleep modifications, sleep disorders and pre-dementia and their impact on the progression of cognitive and behavioral impairment.

## Author Contributions

JT participated in the study concept and design, data acquisition, data analysis and interpretation, interpretation of results, and writing of the manuscript. PS participated in study concept and design, data analysis and interpretation, interpretation of results, writing of the manuscript, and study supervision. CB and MB participated in study concept and design, data acquisition, data analysis and interpretation, interpretation of results, and manuscript revision. HA and J-FD participated in study concept and design and manuscript revision. MR and SH participated in data acquisition and manuscript revision. J-AM-F participated in manuscript revision. PP participated in study concept and design, interpretation of results, writing of the manuscript, and study supervision.

### Conflict of Interest Statement

The authors declare that the research was conducted in the absence of any commercial or financial relationships that could be construed as a potential conflict of interest.

## References

[1] Yaffe K Laffan AM Harrison SL Redline S Spira AP Ensrud KE. Sleep-disordered breathing, hypoxia, and risk of mild cognitive impairment and dementia in older women. JAMA. (2011) 306:613–9. 10.1001/jama.2011.1115 21828324 PMC3600944

[2] Blackwell T Yaffe K Laffan A Ancoli-Israel S Redline S Ensrud KE. Associations of objectively and subjectively measured sleep quality with subsequent cognitive decline in older community-dwelling men: the MrOS sleep study. Sleep. (2014) 37:655–63. 10.5665/sleep.3562 24899757 PMC4044750

[3] Suh SW Han JW Lee JR Byun S Kwon SJ Oh SH. Sleep and cognitive decline: A prospective nondemented elderly cohort study. Ann Neurol. (2018) 83:472–82. 10.1002/ana.25166 29394505

[4] Buzsáki G. Two-stage model of memory trace formation: a role for “noisy” brain states. Neuroscience. (1989) 31:551–70. 10.1016/0306-4522(89)90423-5 2687720

[5] Diekelmann S Born J. The memory function of sleep. Nat Rev Neurosci. (2010) 11:114–26. 10.1038/nrn2762 20046194

[6] Born J Wilhelm I. System consolidation of memory during sleep. Psychol Res. (2012) 76:192–203. 10.1007/s00426-011-0335-6 21541757 PMC3278619

[7] Dudai Y Karni A Born J. The consolidation and transformation of memory. Neuron. (2015) 88:20–32. 10.1016/j.neuron.2015.09.004 26447570

[8] Maingret N Girardeau G Todorova R Goutierre M Zugaro M. Hippocampo-cortical coupling mediates memory consolidation during sleep. Nat Neurosci. (2016) 19:959–64. 10.1038/nn.4304 27182818

[9] Latchoumane CV Ngo HV Born J Shin HS. Thalamic spindles promote memory formation during sleep through triple phase-locking of cortical, thalamic, and hippocampal rhythms. Neuron. (2017) 95:424–35 e6. 10.1016/j.neuron.2017.06.025 28689981

[10] Staresina BP Bergmann TO Bonnefond M van der Meij R Jensen O Deuker L. Hierarchical nesting of slow oscillations, spindles and ripples in the human hippocampus during sleep. Nat Neurosci. (2015) 18:1679–86. 10.1038/nn.4119 26389842 PMC4625581

[11] Klinzing JG Mölle M Weber F Supp G Hipp JF Engel AK. Spindle activity phase-locked to sleep slow oscillations. Neuroimage. (2016) 134:607–16. 10.1016/j.neuroimage.2016.04.031 27103135

[12] Schreiner T Rasch B. Boosting vocabulary learning by verbal cueing during sleep. Cereb Cortex. (2015) 25:4169–79. 10.1093/cercor/bhu139 24962994

[13] Bergmann TO Mölle M Diedrichs J Born J Siebner HR. Sleep spindle-related reactivation of category-specific cortical regions after learning face-scene associations. Neuroimage. (2012) 59:2733–42. 10.1016/j.neuroimage.2011.10.036 22037418

[14] Ferini-Strambi L Galbiati A Marelli S. Sleep microstructure and memory function. Front Neurol. (2013) 4:159. 10.3389/fneur.2013.00159 24130550 PMC3795358

[15] Ulrich D. Sleep spindles as facilitators of memory formation and learning. Neural Plast. (2016) 2016:1796715. 10.1155/2016/1796715 27119026 PMC4826925

[16] Tononi G Cirelli C. Sleep function and synaptic homeostasis. Sleep Med Rev. (2006) 10:49–62. 10.1016/j.smrv.2005.05.002 16376591

[17] Tononi G Cirelli C. Sleep and the price of plasticity: from synaptic and cellular homeostasis to memory consolidation and integration. Neuron. (2014) 81:12–34. 10.1016/j.neuron.2013.12.025 24411729 PMC3921176

[18] Ohayon MM Carskadon MA Guilleminault C Vitiello MV. Meta-analysis of quantitative sleep parameters from childhood to old age in healthy individuals: developing normative sleep values across the human lifespan. Sleep. (2004) 27:1255–73. 10.1093/sleep/27.7.1255 15586779

[19] Bliwise DL Scullin MK Normal aging. In: Kryger M Roth T Dement B, editors. Principles and Practice of Sleep Medicine. 6th ed. Philadelphia: Elsevier (2017). p. 25–38. 10.1016/B978-0-323-24288-2.00003-9

[20] Cajochen C Münch M Knoblauch V Blatter K Wirz-Justice A. Age-related changes in the circadian and homeostatic regulation of human sleep. Chronobiol Int. (2006) 23:461–74. 10.1080/07420520500545813 16687319

[21] Carrier J Land S Buysse DJ Kupfer DJ Monk TH. The effects of age and gender on sleep EEG power spectral density in the middle years of life (ages 20-60 years old). Psychophysiology. (2001) 38:232–42. 10.1111/1469-8986.3820232 11347869

[22] Landolt HP Borbély AA. Age-dependent changes in sleep EEG topography. Clin Neurophysiol. (2001) 112:369–77. 10.1016/S1388-2457(00)00542-3 11165543

[23] Fogel S Martin N Lafortune M Barakat M Debas K Laventure S. NREM sleep oscillations and brain plasticity in aging. Front Neurol. (2012) 3:176. 10.3389/fneur.2012.00176 23248614 PMC3522106

[24] Pace-Schott EF Spencer RM. Sleep-dependent memory consolidation in healthy aging and mild cognitive impairment. Curr Top Behav Neurosci. (2015) 25:307–30. 10.1007/7854_2014_300 24652608

[25] Hu M Zhang P Li C Tan Y Li G Xu D. Sleep disturbance in mild cognitive impairment: a systematic review of objective measures. Neurol Sci. (2017) 38:1363–71. 10.1007/s10072-017-2975-9 28455768

[26] Westerberg CE Mander BA Florczak SM Weintraub S Mesulam MM Zee PC. Concurrent impairments in sleep and memory in amnestic mild cognitive impairment. J Int Neuropsychol Soc. (2012) 18:490–500. 10.1017/S135561771200001X 22300710 PMC3468412

[27] Gorgoni M Lauri G Truglia I Cordone S Sarasso S Scarpelli S. Parietal fast sleep spindle density decrease in Alzheimer's disease and amnesic mild cognitive impairment. Neural Plast. (2016) 2016:8376108. 10.1155/2016/8376108 27066274 PMC4811201

[28] Bliwise DL. Sleep in normal aging and dementia. Sleep. (1993) 16:40–81. 10.1093/sleep/16.1.40 8456235

[29] Rauchs G Schabus M Parapatics S Bertran F Clochon P Hot P. Is there a link between sleep changes and memory in Alzheimer's disease? Neuroreport. (2008) 19:1159–62. 10.1097/WNR.0b013e32830867c4 18596620 PMC2925139

[30] Peter-Derex L Yammine P Bastuji H Croisile B. Sleep and Alzheimer's disease. Sleep Med Rev. (2015) 19:29–38. 10.1016/j.smrv.2014.03.007 24846773

[31] Mander BA Winer JR Jagust WJ Walker MP. Sleep: a novel mechanistic pathway, biomarker, and treatment target in the pathology of Alzheimer's disease? Trends Neurosci. (2016) 39:552–66. 10.1016/j.tins.2016.05.002 27325209 PMC4967375

[32] Latreille V Carrier J Lafortune M Postuma RB Bertrand JA Panisset M. Sleep spindles in Parkinson's disease may predict the development of dementia. Neurobiol Aging. (2015) 36:1083–90. 10.1016/j.neurobiolaging.2014.09.009 25442116

[33] Reda F Gorgoni M Lauri G Truglia I Cordone S Scarpelli S In search of sleep biomarkers of Alzheimer's disease: K-Complexes do not discriminate between patients with mild cognitive impairment and healthy controls. Brain Sci. (2017) 7:E51 10.3390/brainsci7050051 28468235 PMC5447933

[34] De Gennaro L Gorgoni M Reda F Lauri G Truglia I Cordone S. The fall of sleep K-complex in Alzheimer disease. Sci Rep. (2017) 7:39688. 10.1038/srep39688 28045040 PMC5206737

[35] Ladenbauer J Ladenbauer J Külzow N de Boor R Avramova E Grittner U. Promoting sleep oscillations and their functional coupling by transcranial stimulation enhances memory consolidation in mild cognitive impairment. J Neurosci. (2017) 37:7111–24. 10.1523/JNEUROSCI.0260-17.2017 28637840 PMC6705731

[36] Dufouil C Dubois B Vellas B Pasquier F Blanc F Hugon J. Cognitive and imaging markers in non-demented subjects attending a memory clinic: study design and baseline findings of the MEMENTO cohort. Alzheimers Res Ther. (2017) 9:67. 10.1186/s13195-017-0288-0 28851447 PMC5576287

[37] Morris JC. The Clinical Dementia Rating (CDR): current version and scoring rules. Neurology. (1993) 43:2412–4. 10.1212/WNL.43.11.2412-a 8232972

[38] Hugonot-Diner L MMS version consensuelle GRECO. In: La Consultation Engériatrie. Paris: Masson (2001). p. 13–20.

[39] Grober E Buschke H Crystal H Bang S Dresner R. Screening for dementia by memory testing. Neurology. (1988) 38:900–3. 10.1212/WNL.38.6.900 3368071

[40] Tombaugh TN. Trail making test A and B: normative data stratified by age and education. Arch Clin Neuropsychol. (2004) 19:203–14. 10.1016/S0887-6177(03)00039-8 15010086

[41] Petersen RC. Mild cognitive impairment as a diagnostic entity. J Intern Med. (2004) 256:183–94. 10.1111/j.1365-2796.2004.01388.x 15324362

[42] Folstein MF Folstein SE McHugh PR. Mini-mental state. A practical method for grading the cognitive state of patients for the clinician. J Psychiatr Res. (1975) 12:189–98. 10.1016/0022-3956(75)90026-6 1202204

[43] Reitan RM Wolfson D The Halstead-Reitan Neuropsychological Test Battery. Tucson, AZ: Neuropsychology Press (1985).

[44] Johns MW. A new method for measuring daytime sleepiness: the Epworth sleepiness scale. Sleep. (1991) 14:540–5. 10.1093/sleep/14.6.540 1798888

[45] Buysse DJ Reynolds CF Monk TH Berman SR Kupfer DJ. The Pittsburgh Sleep Quality Index: a new instrument for psychiatric practice and research. Psychiatry Res. (1989) 28:193–213. 10.1016/0165-1781(89)90047-4 2748771

[46] Bastien CH Vallières A Morin CM. Validation of the Insomnia Severity Index as an outcome measure for insomnia research. Sleep Med. (2001) 2:297–307. 10.1016/S1389-9457(00)00065-4 11438246

[47] Hatfield CF Herbert J van Someren EJ Hodges JR Hastings MH. Disrupted daily activity/rest cycles in relation to daily cortisol rhythms of home-dwelling patients with early Alzheimer's dementia. Brain. (2004) 127(Pt 5):1061–74. 10.1093/brain/awh129 14998915

[48] Berry RB Brooks R Gamaldo CE Harding SM Lloyd RM Quan SF The AASM Manual for the Scoring of Sleep and Associated Events: Rules, Terminology and Technical Specifications, Version 2.1. Darien, IL: American Academy of Sleep Medicine (2014).

[49] Berthomier C Drouot X Herman-Stoïca M Berthomier P Prado J Bokar-Thire D. Automatic analysis of single-channel sleep EEG: validation in healthy individuals. Sleep. (2007) 30:1587–95. 10.1093/sleep/30.11.1587 18041491 PMC2082104

[50] Schmidt C Collette F Leclercq Y Sterpenich V Vandewalle G Berthomier P. Homeostatic sleep pressure and responses to sustained attention in the suprachiasmatic area. Science. (2009) 324:516–9. 10.1126/science.1167337 19390047

[51] Eichenlaub JB Bertrand O Morlet D Ruby P. Brain reactivity differentiates subjects with high and low dream recall frequencies during both sleep and wakefulness. Cereb Cortex. (2014) 24:1206–15. 10.1093/cercor/bhs388 23283685

[52] Reichert CF Maire M Gabel V Viola AU Götz T Scheffler K. Cognitive brain responses during circadian wake-promotion: evidence for sleep-pressure-dependent hypothalamic activations. Sci Rep. (2017) 7:5620. 10.1038/s41598-017-05695-1 28717201 PMC5514145

[53] Dang-Vu TT Hatch B Salimi A Mograss M Boucetta S O'Byrne J. Sleep spindles may predict response to cognitive-behavioral therapy for chronic insomnia. Sleep Med. (2017) 39:54–61. 10.1016/j.sleep.2017.08.012 29157588

[54] Anderer P Klösch G Gruber G Trenker E Pascual-Marqui RD Zeitlhofer J. Low-resolution brain electromagnetic tomography revealed simultaneously active frontal and parietal sleep spindle sources in the human cortex. Neuroscience. (2001) 103:581–92. 10.1016/S0306-4522(01)00028-8 11274780

[55] Feinberg I Floyd TC. Systematic trends across the night in human sleep cycles. Psychophysiology. (1979) 16:283–91. 10.1111/j.1469-8986.1979.tb02991.x 220659

[56] Latreille V Gaubert M Dubé J Lina JM Gagnon JF Carrier J. Age-related cortical signatures of human sleep electroencephalography. Neurobiol Aging. 76:106–14. 10.1016/j.neurobiolaging.2018.12.012 30710833

[57] Mander BA Rao V Lu B Saletin JM Lindquist JR Ancoli-Israel S. Prefrontal atrophy, disrupted NREM slow waves and impaired hippocampal-dependent memory in aging. Nat Neurosci. (2013) 16:357–64. 10.1038/nn.3324 23354332 PMC4286370

[58] Latreille V Carrier J Gaudet-Fex B Rodrigues-Brazète J Panisset M Chouinard S. Electroencephalographic prodromal markers of dementia across conscious states in Parkinson's disease. Brain. (2016) 139(Pt 4):1189–99. 10.1093/brain/aww018 26912643 PMC5841211

[59] Xie L Kang H Xu Q Chen MJ Liao Y Thiyagarajan M. Sleep drives metabolite clearance from the adult brain. Science. (2013) 342:373–7. 10.1126/science.1241224 24136970 PMC3880190

[60] Ju YE McLeland JS Toedebusch CD Xiong C Fagan AM Duntley SP. Sleep quality and preclinical Alzheimer disease. JAMA Neurol. (2013) 70:587–93. 10.1001/jamaneurol.2013.2334 23479184 PMC3676720

[61] Varga AW Wohlleber ME Giménez S Romero S Alonso JF Ducca EL. Reduced slow-wave sleep is associated with high cerebrospinal fluid Abeta42 levels in cognitively normal elderly. Sleep. (2016) 39:2041–8. 10.5665/sleep.6240 27568802 PMC5070758

[62] Mander BA Marks SM Vogel JW Rao V Lu B Saletin JM. beta-amyloid disrupts human NREM slow waves and related hippocampus-dependent memory consolidation. Nat Neurosci. (2015) 18:1051–7. 10.1038/nn.4035 26030850 PMC4482795

[63] Lucey BP Bateman RJ. Amyloid-beta diurnal pattern: possible role of sleep in Alzheimer's disease pathogenesis. Neurobiol Aging. (2014) 35(Suppl. 2):S29–34. 10.1016/j.neurobiolaging.2014.03.035 24910393

[64] Djonlagic I Aeschbach D Harrison SL Dean D Yaffe K Ancoli-Israel S. Associations between quantitative sleep EEG and subsequent cognitive decline in older women. J Sleep Res. (2018) e12666. 10.1111/jsr.12666 29508460 PMC7025429

[65] Ujma PP Halász P Simor P Fabó D Ferri R. Increased cortical involvement and synchronization during CAP A1 slow waves. Brain Struct Funct. (2018) 223:3531–42. 10.1007/s00429-018-1703-4 29951916

[66] Hoedlmoser K Heib DP Roell J Peigneux P Sadeh A Gruber G. Slow sleep spindle activity, declarative memory, and general cognitive abilities in children. Sleep. (2014) 37:1501–12. 10.5665/sleep.4000 25142558 PMC4153050

[67] Geiger A Huber R Kurth S Ringli M Jenni OG Achermann P. The sleep EEG as a marker of intellectual ability in school age children. Sleep. (2011) 34:181–9. 10.1093/sleep/34.2.181 21286251 PMC3022938

[68] Bang JW Khalilzadeh O Hämäläinen M Watanabe T Sasaki Y. Location specific sleep spindle activity in the early visual areas and perceptual learning. Vision Res. (2014) 99:162–71. 10.1016/j.visres.2013.12.014 24380705 PMC4041809

[69] Haba-Rubio J Marti-Soler H Tobback N Andries D Marques-Vidal P Waeber G. Sleep characteristics and cognitive impairment in the general population: the HypnoLaus study. Neurology. (2017) 88:463–9. 10.1212/WNL.0000000000003557 28039311

[70] Martin N Lafortune M Godbout J Barakat M Robillard R Poirier G. Topography of age-related changes in sleep spindles. Neurobiol Aging. (2013) 34:468–76. 10.1016/j.neurobiolaging.2012.05.020 22809452

